# Selenium speciation-dependent cancer radiosensitization by induction of G2/M cell cycle arrest and apoptosis

**DOI:** 10.3389/fbioe.2023.1168827

**Published:** 2023-03-22

**Authors:** Shiqing Nie, Xin He, Zhiting Sun, Yan Zhang, Ting Liu, Tianfeng Chen, Jianfu Zhao

**Affiliations:** Department of Oncology of the First Affiliated Hospital, Jinan University, Guangzhou, Guangdong, China

**Keywords:** selenium, non-small cell lung cancer, radiosensitivity, cell cycle, apoptosis, ROS

## Abstract

**Introduction:** Radiation therapy has Q6long been a routine and effective treatment for non-small cell lung cancer (NSCLC), but the radioresistance and side effects have limited its application. In recent years, the superiority showed by trace element selenium in tumor radiotherapy sensitization has received wide attention. However, different forms of selenium compounds exhibit different chemical properties and their mechanisms of action on tumors may be different.

**Methods:** Human non-small cell lung cancer SPC-A1 cells were studied. Drug toxicity was detected by MTT assay. The selenium content absorbed in vitro at different time points was detected by ICP-MS. Colony formation were conducted to observe the radiosensitization effect of different selenium compounds on SPC-A1 cells, and to compare the proliferation ability of SPC-A1 cells treated by radiation alone and radiation combined with different selenium compounds. Cell migration was detected by cell scratch assay. The changes of cell cycle and apoptosis were detected by flow cytometry. DCFH-DA fluorescent probe was used to detect the effects of different selenium compounds combined with X-ray on ROS production.

**Results:** In this study, these four representative selenium compounds all have a certain ability to enhance the ability of radiotherapy to inhibit tumor cell proliferation and migration, and the mechanism may be related to blocking cell cycle in G2/M phase, activating the caspase cascade and reducing intracellular ROS levels to induce tumor cell apoptosis. Among them, -2-valent organic selenium has the most obvious effect, mainly inhibits cell migration, and induces early apoptosis by activating a large number of caspase-3, and arrest the cell cycle in S phase and G2/M phase. 0-valent selenium nanoparticles mainly arrest the cell cycle in G2/M phase. +4-valent inorganic selenium exerts its antitumor effects primarily by inhibiting tumor cell migration and inducing early apoptosis of tumor cells.

**Discussion:** In this paper, the antitumor effects of four different forms of selenium compounds combined with X-rays on SPC-A1 cells were investigated, and their inhibitory effects on the proliferation and migration of cancer cells and their mechanisms were examined. We found that the radiosensitizing effect of selenium on NSCLC was closely related to its selenium form through the study of the sensitizing effect of different kinds of selenium compounds on radiotherapy.

## 1 Introduction

Lung cancer is one of the cancers with a high mortality rate worldwide, with a 5-year survival rate of less than 20% (Wu et al., 2019; Bade & Dela Cruz, 2020; Sung et al., 2021). Non-small cell lung cancer (NSCLC) accounts for about 80%–85% of all lung cancers, but its low early diagnosis rate makes it easy for patients to miss the best surgical treatment time (Duma et al., 2019; Goebel et al., 2019), so radiotherapy and chemotherapy have become the main treatment for advanced NSCLC. Radiation therapy (RT), as a routine treatment and an important adjunct to lung cancer, can effectively kill most of the cells in the tumor and inhibit the growth of the tumor by directly or indirectly damaging the DNA double-strand (De Ruysscher et al., 2019; Bade & Dela Cruz, 2020). However, this treatment has some limitations because the anatomical division between normal and malignant tumor tissues is not clear, and when using X-rays to damage cancer cells, damage to normal cells is inevitable (Durante et al., 2017; Zhong et al., 2021), cause radioactive inflammation. Therefore, in recent years, A class of drugs that enhance the efficacy of radiation therapy to tumors has been developed, called radiosensitizers. Previous report demonstrated that the combination of paclitaxel and radiotherapy in the treatment of NSCLC significantly increased the radiotherapy sensitivity of NSCLC (Dukaew et al., 2020). Celecoxib and afatinib work together to enhance the radiosensitivity of A549 cells by modulating the cell cycle (Zhang et al., 2021). Osimertinib sensitizes radiotherapy by arresting the cell cycle in a concentration- and time-dependent manner (Wang et al., 2019). But the main disadvantage of these radiosensitizers is their inherent cytotoxicity and side effects. In order to solve the thorny problems of dose toxicity of radiotherapy and systemic side effects of chemotherapy, it is necessary to find highly efficient and low-toxic radiotherapy sensitizers.

Selenium (Se), as a trace element necessary to maintain normal life activities of human body, has important physiological functions and a wide range of pharmacological effects (Buntzel et al., 2010; Handa et al., 2020; Hariharan & Dharmaraj, 2020; Razaghi et al., 2021). In recent years, a large number of studies have reported its role in tumor diagnosis and treatment, including various forms of selenium compounds such as inorganic selenium (Ganash, 2021), organic selenium (Gandin et al., 2018; Chuai et al., 2021), selenium metal complexes (Lai et al., 2019), and nano-selenium (Yu et al., 2015; Liu et al., 2021; Jiao et al., 2022). For example, selenium nanoparticles modified by lentinan (LET-SeNPs) can enhance the anti-prostate cancer cell activity of zoledronic acid (ZOL) (An & Zhao, 2021). Zhang et al. (2018) found that combined treatment of polyethylene glycol nano-selenium (PEG-SeNPs) and X-rays can increase tumor cell apoptosis by activating effector caspase-3 and its downstream targets in a concentration-dependent manner. 5-nitrobenzo [c][1,2,5]selenadiazole (SeD) can significantly enhance the sensitivity of human cervical cancer cells to X-rays and cause G2/M cycle arrest of tumor cells (He et al., 2015). In the study of selenium ruthenium complex, it was found that the introduction of Se can greatly improve the antitumor and antiangiogenic effects of the complex, and can enhance the sensitivity of tumor cells to radiotherapy, and significantly reduce the toxicity of the complex to normal cells (Zhao et al., 2018). Wu et al. (2013) found that nano selenium modified with PRW (Polyporus rhinocerus water-soluble polysaccharide–protein complexes) has an antitumor effect against human lung adenocarcinoma cell A549 cells by inducing apoptosis and G2/M phase arrest. L-Se-methylselenocysteine (L-SeMC/SeMC) can synergize with chemotherapy drugs to increase ROS content for apoptosis in lung cancer cells (Ma et al., 2021). SeNPs@LET with the enhancing immune function improve the anti-tumor ability of lung cancer patients by activating immune cells (Song et al., 2021). Therefore, we can say that Se plays an important role in chemoprophylaxis, treatment, radiotherapy sensitization and other aspects of tumor, and is one of the most potential new high-efficiency and low-toxicity radiotherapy sensitizer materials. So far, there have been few reports of selenium compounds acting on lung cancer, and has no compared the antitumor activity of different valence selenium compounds.

Therefore, we can make wild guesses, selenium compounds with different valence states can kill tumor cells through different ways to enhance the radiotherapy sensitivity of tumor cells. In this study, the radiosensitizing effects of different selenium compounds on NSCLC were compared, and the selenium compounds most suitable for lung cancer radiosensitizing agent were screened out. This study provides a scientific theoretical basis for the further application of selenium compounds into the clinical radiotherapy of NSCLC.

## 2 Materials and methods

### 2.1 Cell culture and cell survival test

SPC-A1 lung cancer cells were purchased from American Type Culture Collection (ATCC) and cultured in a cell incubator with 37°C, 95% relative humidity and 5% CO_2_ concentration. Nutrition came from DMEM medium containing fetal bovine serum (10%), penicillin (100 units/mL) and streptomycin (50 units/mL). Cells in the logarithmic growth phase were used in each cell experiment. Firstly, SPC-A1 cells were seeded in 96-well plates and incubated for 24 h, and different concentrations of different selenium compounds were added respectively for 4 h. Then, cell culture plates were irradiated with 4 Gy and 8 Gy X-rays, respectively, and incubated for 72 h. Finally, cell viability was determined by MTT assay and the half inhibitory concentration (IC50) of the drug on cells was calculated.

### 2.2 Colony formation

After SPC-A1 cells were seeded in 6-well plates for 72 h, they were pretreated with different selenium compounds at different concentrations for 4 h, followed by X-irradiation at 2 or 4 Gy. And cultured at 37°C for 10 days to observe the formation of clones by naked eye. After washing three times with cold PBS, they were fixed with 4% paraformaldehyde for 1 h at room temperature, and then stained with 0.25% methyl violet for 20–30 min. Finally, wash three times with PBS, and take pictures after natural air-drying. Assess the survival fraction of clones.

### 2.3 Cellular uptake

SPC-A1 cells in logarithmic growth phase were seeded in 10 cm cell culture dishes (cell density: 6 × 10^5^ cells/mL, 10 mL/dish). After cell adherence for 24 h, the cells were incubated with Na_2_SeO_3_ (final concentration 8 μM), SeD (final concentration 8 μM), SeC (final concentration 80 μM) and LET-SeNPs (final concentration 80 μM) for 0, 1, 2, 4 and 8 h, respectively. Then, the cells were washed with PBS and collected, the supernatant was discarded, and cell precipitation was nitrified. Finally, we measured the selenium content in the solution by inductively coupled plasma mass spectrometry (ICP-MS), and analyzed the uptake of various selenium compounds by SPC-A1 cells at different time points.

### 2.4 Wound healing experiment

SPC-A1 cells (density: 2.5 × 10^5^ cells/mL, 2 mL) were inoculated into 6-well plates. After the cells have adhered, use a pipette tip to gently draw two lines at the bottom of the confluent well, and wash off the fallen cells with PBS. Take pictures. Then, serum-free medium mixed with drugs was added, and the 6-well plate was placed in an incubator for 24 h. Take pictures again. Calculate cell mobility based on the movement of cells in the photo.

### 2.5 Cell cycle

The cycle distribution of cells can be detected by flow cytometry. (An & Zhao, 2021). SPC-A1 cells were pretreated with different selenium compounds for 4 h, followed by irradiating with 8 Gy. After 72 h, the culture medium in the cell culture dish was collected, the cells were washed with PBS, and then treated with 0.25% trypsin. The supernatant and cell suspension were collected in a 15 mL centrifuge tube, centrifuged (1,500 rpm, 5 min) to collect the pellet. It was then fixed with pre-chilled 75% ethanol overnight at −20°C. The next day, taken it out and centrifuged (1,500 rpm, 5 min), washed with PBS once to remove alcohol, then centrifuged, and stained with 500 μL PI staining solution for 30 min in the dark. Finally, samples were analyzed with a Beckman Coulter flow cytometer, and the collected cell cycle results were analyzed with CytExpert software.

### 2.6 Cell apoptosis

Annexin Ⅴ/PI apoptosis detection kit was used to detect the apoptosis level of cells (Zhao et al., 2018). After staining, the apoptosis level of cells was detected by flow cytometry. By analyzing the proportion of early apoptosis and late apoptosis in each group, the difference of apoptosis induced by different selenium compounds combined with radiotherapy was compared.

### 2.7 Determination of intracellular ROS

DCFH-DA fluorescent probe was used to detect the effects of different selenium compounds combined with X-rays on ROS production (Kim & Xue, 2020). SPC-A1 cells (density: 2.5 × 10^5^ cell/mL, 100 µL) were inoculated in a 96-well plate, incubated in an incubator for 24 h, and different selenium compounds with different concentrations (100 µL) were added and incubated for 4 h, and then the cells was irradiated with a dose of 8 Gy. Aspirated the medium in each well and added DCFH-DA 10 µM (100 µL) for 30 min in the dark for staining. The excitation wavelength was set to 488 nm and the emission wavelength was set to 525 nm, and the fluorescence intensity of each well was detected by a fluorescence microplate reader for 2 h. Simultaneously monitored the DCF fluorescence intensity in the cells in real time with a fluorescence microscope.

### 2.8 Caspase-3 activity assay

The fluorescence intensity of caspase-3 substrate can be used to detect the activation of intracellular caspases. All dishes were incubated in an incubator for 24 h. Different concentrations of Na_2_SeO_3_, SeD, SeC, and lentinan nano-selenium (LET-SeNPs) were added for pretreatment for 4 h, irradiated with 8 Gy X-rays and continued to be incubated for 72 h. The cellular proteins in the above dishes were collected with BCA kit. Mixed an equal volume of protein sample with 2 × caspase substrate assay solution and incubated at room temperature for 2 h in the dark. Set the excitation wavelength at 365 nm and the emission wavelength at 450 nm, read the fluorescence value of each well solution with a fluorescence microplate reader, and calculated the relative activity of caspase.

### 2.9 Statistical analysis

All experiments in this subject were repeated at least three times, and the results were expressed as mean ± standard deviation (mean ± SD), and statistical analysis was performed using the statistical software SPSS 13.0. Two-tailed t-tests were utilized between two groups, and multiple comparisons were utilized between multiple groups, with *p < 0.05 considered significant and **p < 0.01 considered highly significant.

## 3 Results and discussion

### 3.1 *In vitro* radiosensitization activity of different selenium compounds

**FIGURE 1:**
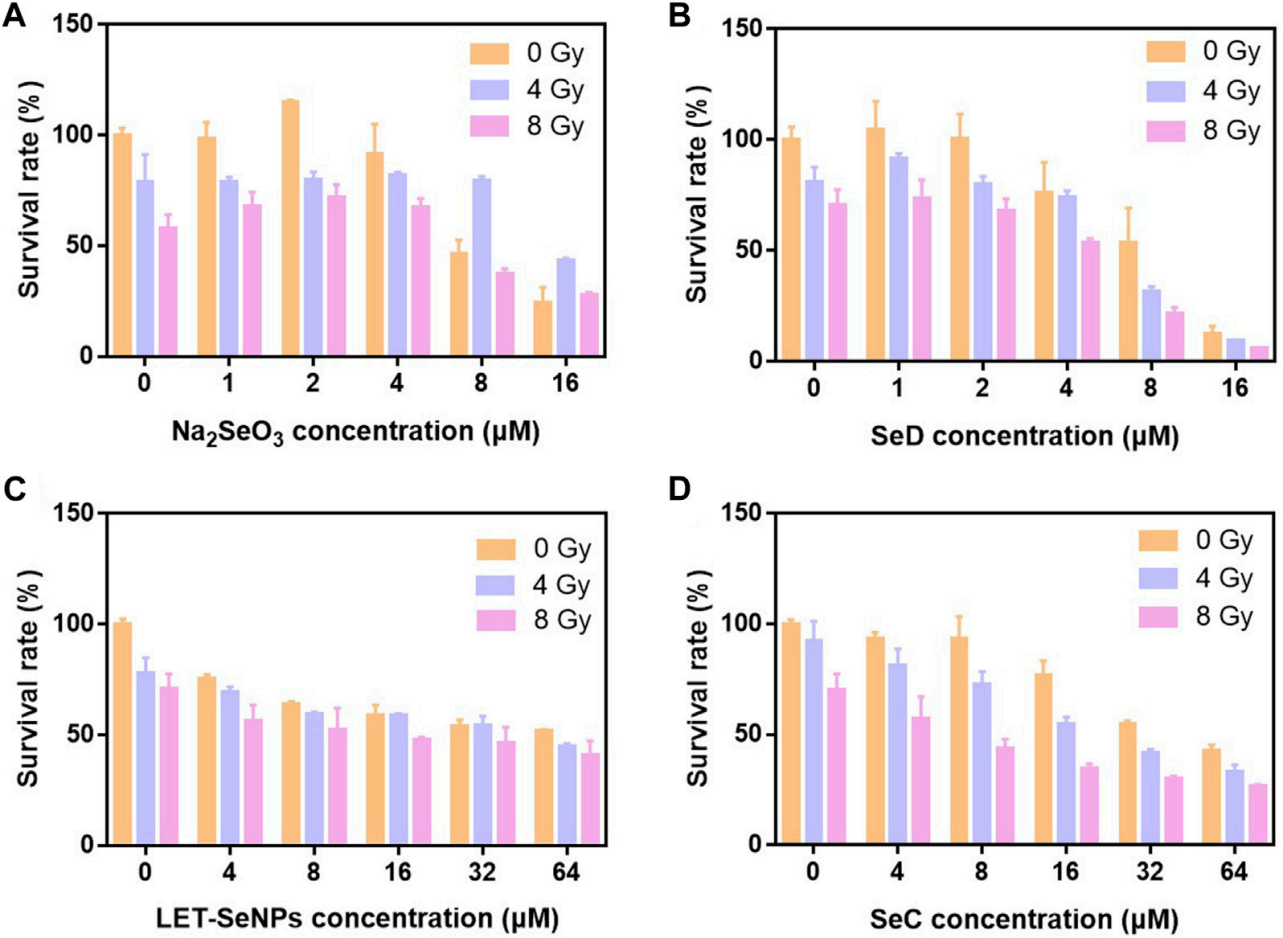
Histogram of cell survival of different selenium compounds under X-ray action. **(A–D)** Cell viability of SPC-A1 cells after treatment with different selenium compounds combination with X-rays for 72 h.

In order to study the effect of different valence of selenium compounds on tumor radiotherapy sensitization. We used Na_2_SeO_4_, Na_2_SeO_3_, LET-SeNPs, chitosan nano-selenium (CS-SeNPs), PEG-SeNPs, selenomethionine (SeM), SeC, SeD and Ebselen to evaluate their antitumor activities *in vitro*. First, we detected the effect of different doses of X-rays on the survival rate of SPC-A1 cells. The results showed that the proliferation of SPC-A1 cells was inhibited to different degrees under different doses of X-rays irradiation. Even under the highest dose of 8 Gy, the survival rate of SPC-A1 cells was still higher than 70%. These results indicated that SPC-A1 cells were tolerant to radiotherapy (Supplementary Figure S1). Then, MTT assay was used to detect the anti-tumor activity of different selenium compounds combined with X-rays *in vitro*, and the IC50 of different selenium compounds on SPC-A1 cells was studied. As shown in Figure 1 and Supplementary Figures S2, S3, among the 9 selenium compounds, except Na_2_SeO_4_ and SeM, the other seven showed different antitumor activities in general, and the cytotoxicity increased with the increase of selenium content. However, it is interesting that the various drugs show the effect of promoting cell proliferation when the selenium concentration is less than 4 μM, while the cytotoxicity is only shown when the selenium concentration is higher than 4 μM. Through the analysis of IC50 of SPC-A1 cells treated with different selenium compounds alone and SPC-A1 cells treated with X-rays combined (Supplementary Table S1), it was proved that selenium compounds did enhance the killing effect of X-rays on tumor cells. Besides, the anti-tumor activities of other selenium compounds except Na_2_SeO_4_ and SeM were improved under the action of X-rays. To sum up, the histogram of the survival rate of SPC-A1 cells after four kinds of selenium compounds, LET-SeNPs, Na_2_SeO_3_, SeC and SeD combined with radiotherapy, we selected these 4 selenium compounds to further explore their ways of enhancing the effect of radiotherapy.

### 3.2 Cellular uptake of different selenium compounds

**FIGURE 2:**
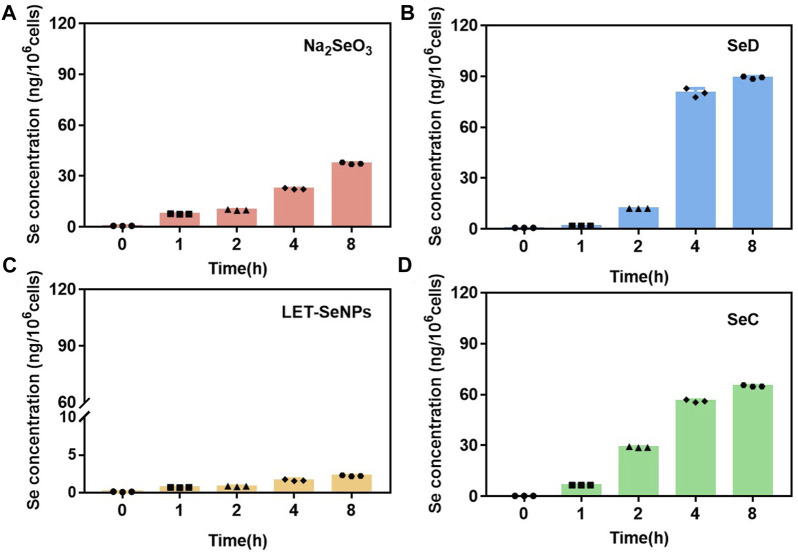
Cellular uptake of different selenium compounds (by Se content). **(A)** Na_2_SeO_3_ and **(B)** SeD at different time points (0, 1, 2, 4 and 8 h) at 8 μM. **(C)** LET-SeNPs and **(D)** SeC at different time points (0, 1, 2, 4 and 8 h) at 80 μM.

It is well known that whether a drug can be absorbed and its half-life in the body are important indicators affecting the efficacy of a drug (Smith et al., 2018). Therefore, we quantified the selective uptake levels of LET-SeNPs, Na_2_SeO_3_, SeC and SeD in SPC-A1 cells by using ICP-MS to detect the intracellular selenium content into SPC-A1 cells. From Figure 2, we can clearly see that the accumulation of these four drugs in SPC-A1 cells showed a time-dependent, among them, the intracellular selenium content of SeD increased rapidly within 2 h, from the initial 0.65 ng/10^6^ cells to 80.25 ng/10^6^ cells. However, although the uptake of LET-SeNPs in SPC-A1 cells also increased with time, the uptake was very low, and the intracellular selenium content was only 1.67 ng/10^6^ cells after 8 h of exposure. Looking at the other two drugs, the absorption amount and absorption speed of SeC and Na_2_SeO_3_ were similar, and there was no statistically significant difference. From the results, we can see that SeD with the most significant anti-tumor effect is absorbed by SPC-A1 cells at the fastest speed and the largest amount during the same action time, and has played a greater toxic effect. In conclusion, the toxic effects of selenium-containing compounds on SPC-A1 cells are proportional to their uptake by cells.

### 3.3 Different selenium compounds combinated with X-rays to inhibit the proliferation of SPC-A1 cells

**FIGURE 3:**
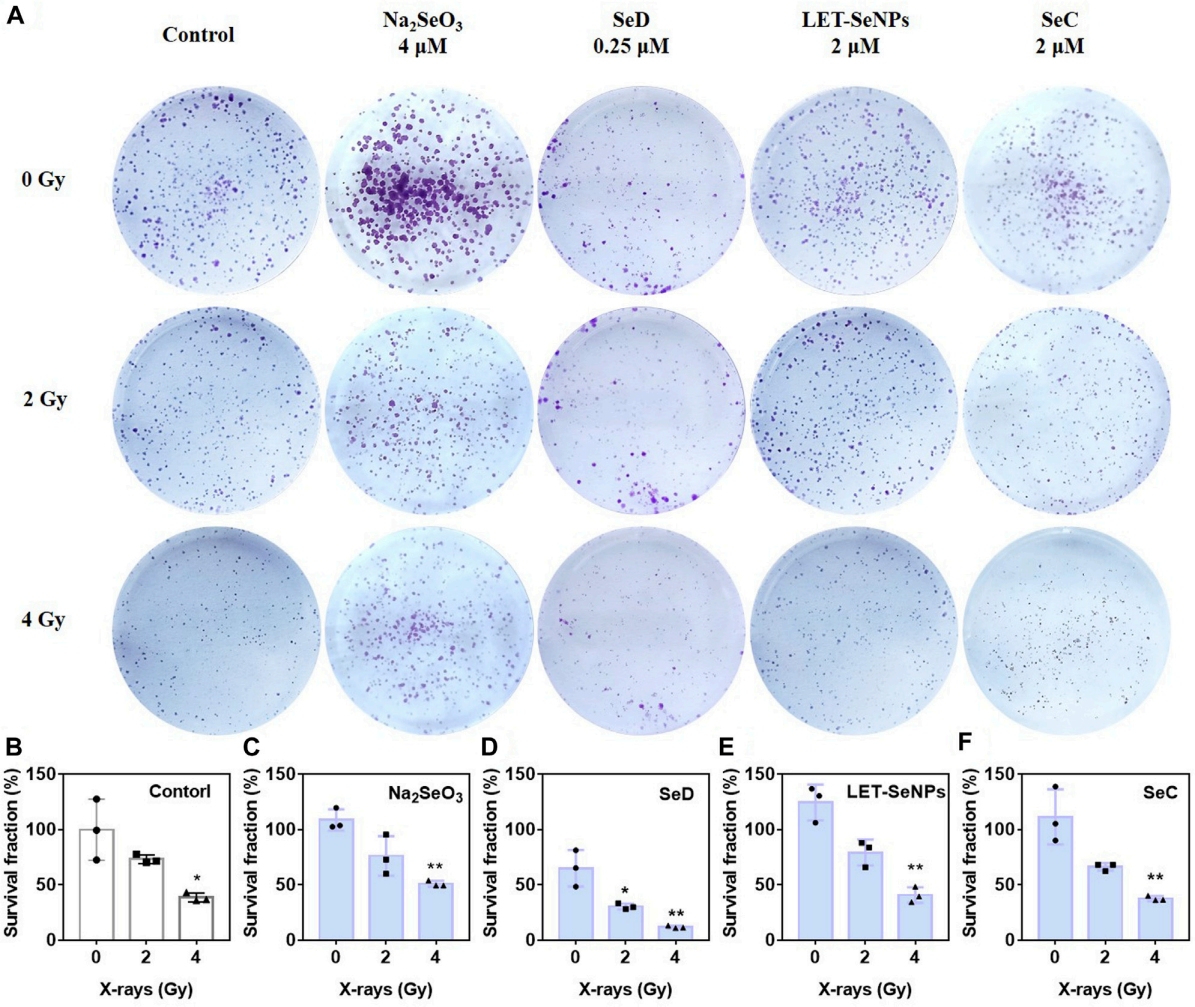
Different selenium compounds inhibited the clonogenesis of SPC-A1 cells. **(A)** Clonogenic assay treated by different selenium compounds with or without X-rays for 14 d in SPC-A1 cells. **(B–F)** Clonogenic formation a bar chart of SPC-A1 cells under the co-treatment of different selenium compounds and X-rays (0–4 Gy). Values expressed were means ± SD of triplicates. *p < 0.05 considered significant and **p < 0.01 considered highly significant.

Colony formation can detect the clonogenesis ability of adherent cells and reflect their colony dependence and proliferation ability. As shown in Figure 3, under the irradiation of X-rays alone, the clone formation rate gradually decreased with increasing radiotherapy dose, where the clone formation rate in the control group decreased to 39.4% under the effect of 4 Gy. In the drug-acting group, the most pronounced decline was SeD, where the clonal formation rate in the SeD group alone had dropped to 66.69% without X-rays irradiation. When irradiated with 4 Gy, the clonal formation rate was directly as low as 12.08%. Under the action of low-dose selenium compounds, compared with the control group, the other three drugs except SeD did not significantly inhibit cell clonal formation with or without X-rays irradiation. Overall, SeD has the most obvious radiosensitization effect among the four selenium compounds, and its concentration of action is lower than that of other drugs, while playing a significant role in inhibiting tumor cell adherent and proliferation.

### 3.4 Different selenium compounds inhibited the migration of SPC-A1 cells

**FIGURE 4:**
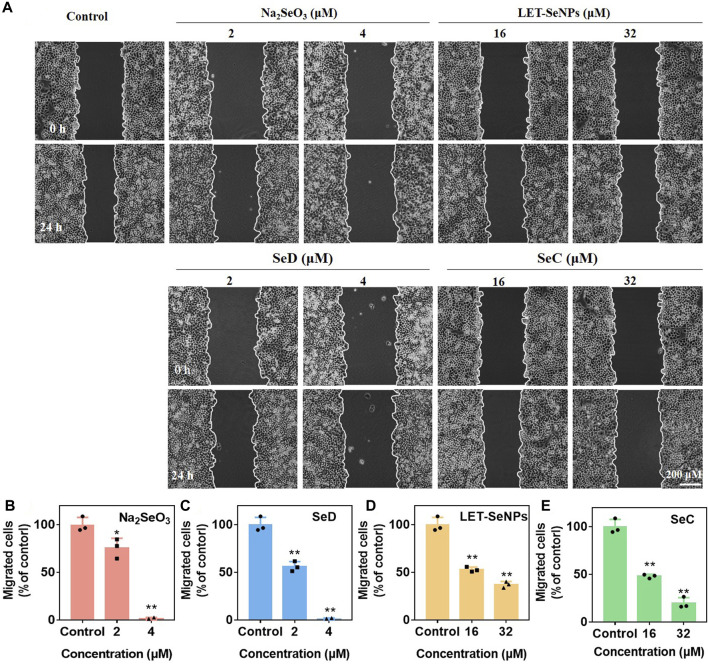
Inhibition of cancer cell migration of different selenium compounds. **(A)** Wound healing assay effects of different selenium compounds on SPC-A1 cells. **(B–E)** Quantitative analysis of the migrated SPC-A1 cells under the influence of different selenium compounds by manual counting. *p < 0.05 considered significant and **p < 0.01 considered highly significant.

In addition, malignant tumor metastasis is the main reason for the failure of advanced tumor treatment. Tumor cells have low homogeneous adhesion and strong motility, and can transfer to other parts of the body along with blood vessels or lymphatic vessels, resulting in tumor metastasis. In order to understand the migration ability of SPC-A1 cells under the action of drugs, we studied and compared the migration rates of SPC-A1 lung cancer cells under the action of different drugs through wound healing experiments. As shown in Figure 4A, the migration ability of SPC-A1 cells decreased to varying degrees after different concentrations of different drugs acted for 24 h, which were significantly lower than those of the control group. As can be seen from Figures 4B–E, different kinds of selenium compounds inhibited the migration ability of SPC-A1 cells in a dose-dependent manner. When the four drugs were compared with each other at low concentrations, the migration ability of cells was the strongest after Na_2_SeO_3_ treatment for 24 h, and there was no statistical significance in the degree of decrease in cell migration ability after the other three drugs were used (*p* > 0.01). With the increase of drug concentration, it can be seen that the migration ability of SPC-A1 cells was significantly reduced after the action of Na_2_SeO_3_ (4 μM) and SeD (4 μM) for 24 h, and it could even be said that the migration ability was completely lost. And SeC (32 μM) inhibited cell migration more than LET-SeNPs (32 μM). Comprehensive comparison, SeD inhibited the migration ability of SPC-A1 cells stronger, and the required drug concentration was lower, which was obviously better than the other three selenium compounds.

### 3.5 Effects of different selenium compounds combinated with X-rays on SPC-A1 cell cycle

**FIGURE 5:**
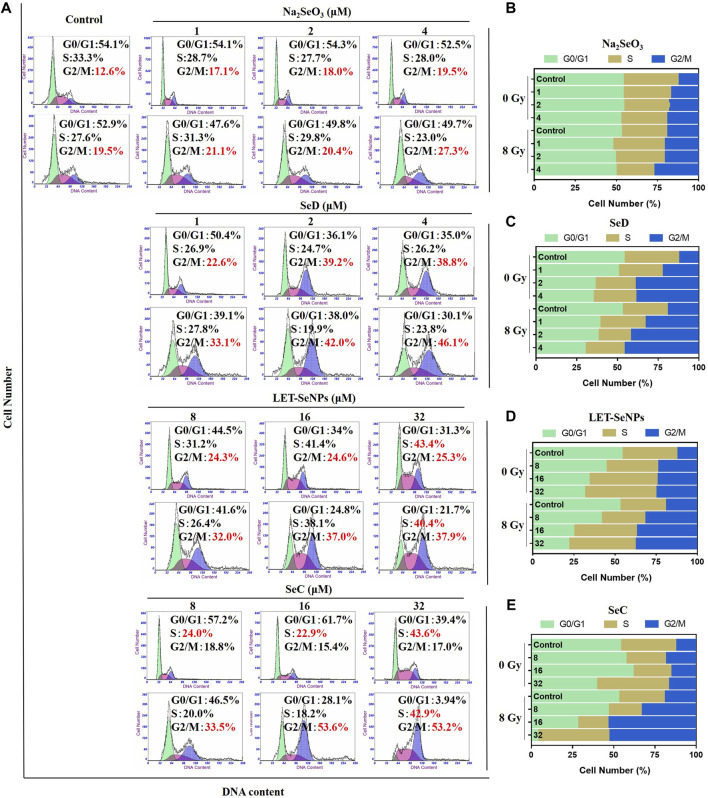
Cell cycle distribution analysis after treatment with different selenium compounds. **(A)** Flow cytometry cycle distribution of SPC-A1 cells treated with different selenium compounds (with or without X-rays) for 72 h **(B–E)** Are histogram of cell cycle distribution after treatment with Na_2_SeO_3_, SeD, LET-SeNPs and SeC, respectively.

Studies have shown that cell cycle arrest affects the occurrence and development of tumors (Hung et al., 2021). In order to further study the effects of different selenium compounds on the intrinsic mechanism of SPC-A1 cells, we used flow cytometry to determine whether different selenium compounds could inhibit tumor cell proliferation by affecting the SPC-A1 cell cycle. As can be seen from Figure 5, SPC-A1 cells were treated with various selenium compounds at their respective effective selenium concentrations for 72 h, and the cell cycle changed in both the radiotherapy group and the non-radiotherapy group. In the control group, the G2/M phase of cells increased from 12.6% without radiotherapy to 19.5% after radiotherapy, indicating that radiation mainly blocked the cell cycle in G2/M phase. In the drug group, Na_2_SeO_3_ alone increased the G2/M phase of cells from 12.6% to 19.5%, and G2/M phase increased from 19.5% to 27.3% when combined with X-rays. The G2/M phase increased to 38.8% when treated with SeD alone and 46.1% after treated with radiotherapy. These results indicated that these two drugs mainly acted on G2/M phase of cell cycle arrest. Meanwhile, LET-SeNPs and SeC inhibited SPC-A1 cell cycle not only in G2/M phase, but also in S phase. The changes of SPC-A1 cells in S phase were more obvious than those in G2/M phase with LET-SeNPs, regardless of the effect of X-rays. In general, the effects of different selenium compounds on the cell cycle of SPC-A1 showed a dose effect, and radiotherapy mainly blocked the cell cycle in the G2/M phase. Na_2_SeO_3_, SeD, LET-SeNPs and SeC enhanced the effect of radiotherapy on G2/M phase arrest to varying degrees, and SeC was the best, followed by SeD.

### 3.6 Different selenium compounds combinated with X-rays to induced SPC-A1 cells apoptosis

**FIGURE 6:**
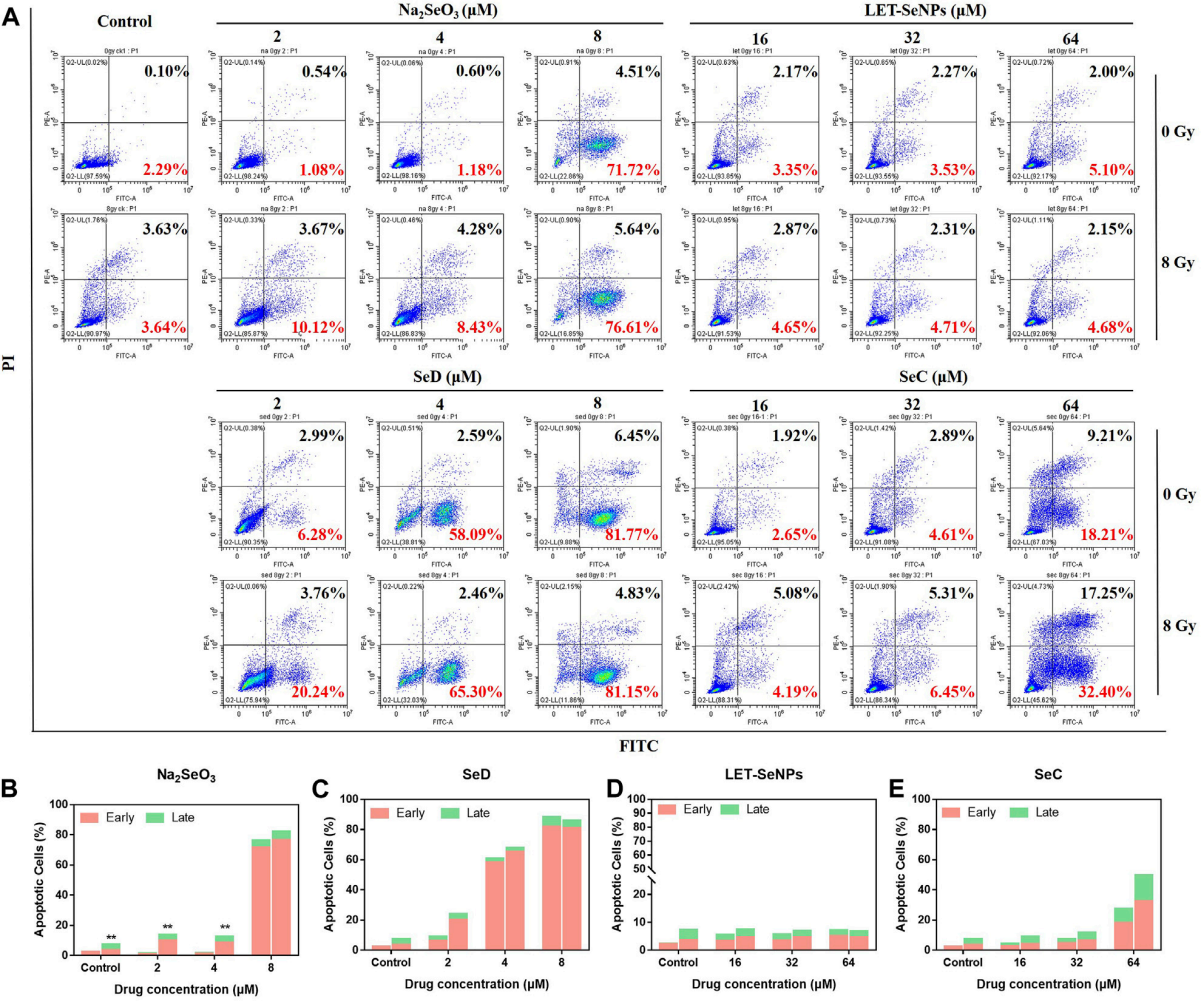
Induction of cell apoptosis after treatment with different selenium compounds. **(A)** Flow cytometry apoptosis profile of SPC-A1 cells treated with different selenium compounds (with or without X-rays) for 72 h **(B–E)** Are histogram of apoptosis distribution after treatment with Na_2_SeO_3_, SeD, LET-SeNPs and SeC, respectively.

The inhibitory effect of drugs on tumor cells can exert its anti-tumor effect not only by affecting the tumor cell cycle, but also by causing tumor cell apoptosis. Different selenium compounds can induce apoptosis of SPC-A1 lung cancer cells. Apoptosis was increased in a dose-dependent manner when treated with different concentrations of Na_2_SeO_3_, SeD, LET-SeNPs and SeC in combination with X-rays (Figure 6). Among them, Na_2_SeO_3_ and SeD caused a small amount of apoptosis at low doses, but a large amount of apoptosis at a concentration of 8 μM. Cell apoptosis increased from 2.39% to 88.22% when SeD drugs acted alone. However, the apoptosis rate after radiotherapy is not much different from that of single drug. It may be because the drug concentration is too high to make the drug effect dominate, and the effect of radiotherapy is not obvious. The apoptosis rate of SPC-A1 cells treated with SeC alone or combined with X-rays for 72 h was dose-dependent, and early apoptosis and late apoptosis occurred at the same time. Compared with other drugs, the late apoptosis accounted for a large proportion. However, the apoptosis rate of LET-SeNPs (64 μM) was not different from that of the control group. In conclusion, Most of the cell apoptosis induced by the combined action of different selenium compounds and X-rays was higher than that caused by drug alone. Among them, LET-SeNPs induced the least apoptosis, and the combined action of SeD, SeC and Na_2_SeO_3_ combined with X-rays could effectively increase cell apoptosis, and the effect strength was SeD > Na_2_SeO_3_>SeC.

### 3.7 Different selenium compounds combinated with X-rays to reduced ROS production in SPC-A1 cells

**FIGURE 7:**
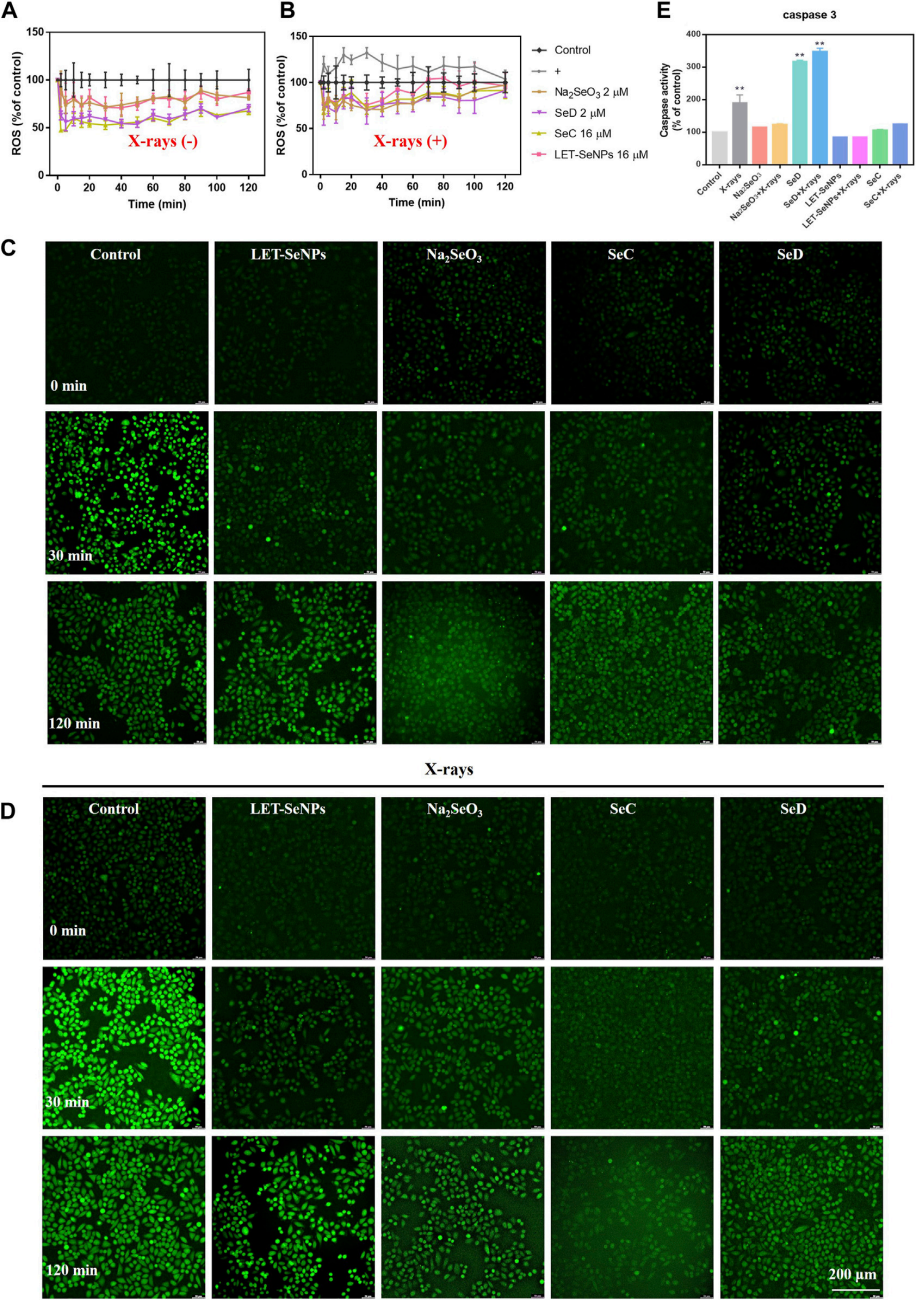
Different selenium compounds combination with X-rays changes intracellular ROS levels and quantitative analysis of caspase activation triggered by selenide and X-rays. **(A)** ROS changes after interaction of different selenium compounds. **(B)** ROS changes after interaction of different selenium compounds and X-rays. “(+)” means to receive X-rays irradiation. **(C)** Fluorescence imaging of ROS generation in SPC-A1 cells after the incubation of different selenium compounds for indicated times using a DCF probe. Original magnification: ×10. **(D)** Fluorescence imaging of ROS generation in SPC-A1 cells after the incubation of different selenium compounds and X-rays (8 Gy) for indicated times using a DCF probe. Original magnification: ×10. **(E)** Values expressed were means ± SD of triplicates. *p < 0.05 considered significant and **p < 0.01 considered highly significant.

ROS has long been associated with cancer, with most tumor cells produce higher levels of ROS than normal cells. The increase may be due to the reduction of free radical scavenging enzymes (Cui et al., 2018), increased glucose metabolism (Warburg effect) (DeBerardinis & Chandel, 2020), increased fatty acid oxidation (Sun & Denko, 2014) and so on. In most studies, elevated ROS levels are considered carcinogenic, and high levels can damage DNA double strands, proteins, and lipids, increasing genetic mutations and promoting tumor formation (Nakamura & Takada, 2021; Renaudin, 2021; Shah & Rogoff, 2021). But there are also studies showing toxic levels of ROS produced in cancer have antitumor effects, leading to increased oxidative stress and inducing tumor cell death (Nogueira et al., 2008). In order to further study the radiotherapy sensitization mechanism of different selenium compounds, we used DCF-DA probe to detect ROS produced by Na_2_SeO_3_, SeD, LET-SeNPs and SeC combined with X-rays or not. The results are shown in Figure 7A and Supplementary Figure S4A, the effect of the drug alone downregulated the level of intracellular ROS, and the intracellular ROS decreased to the lowest level within 30 min of drug action, and then gradually increased. The ROS levels in cells treated with SeD and SeC were basically the same, and there was little difference in ROS levels after treated with Na_2_SeO_3_ and LET-SeNPs. The most significant decrease was in SPC-A1 cells treated with SeC alone, where the ROS level in the first 10 min decreased to the lowest 47.4%. As shown in Figure 7B and Supplementary Figure S4B, X-rays alone can cause ROS levels to rise, inducing ROS production within 30 min and reaching a peak of 132.13%. In contrast, individual drug effects significantly downregulated ROS levels. ROS levels were slightly higher when Na_2_SeO_3_, SeD, LET-SeNPs and SeC combined with X-rays compared with drugs alone. Combined with the real-time dynamic fluorescence images captured under the fluorescence microscope (Figures 7C, D), the generation of fluorescence amount is consistent with the ROS change curves of Figures 7A, B, and the ROS generated in cells after X-rays irradiation is significantly more than that generated in cells after drug alone. Based on the above experimental results, we speculate that Na_2_SeO_3_, SeD, LET-SeNPs, and SeC sensitize X-rays to induce apoptosis by scavenging oxidative free radicals in cells and then causing redox imbalance.

### 3.8 Changes intracellular caspase-3 activity induced by different selenium compounds

Caspase is a cysteine protease family with 14 members that play a key role in programmed cell death and inflammation (Van Opdenbosch & Lamkanfi, 2019). Among them, caspase-3 is a typical executor of apoptosis. After being activated by caspase-8 or caspase-9, it cleaves many other functional key proteins in the cell, resulting in apoptosis. Its activation heralds the start of the executive phase of apoptosis. In this experiment, we used fluorochrome substrate colorimetry to measure the changes of caspase-3 activity in SPC-A1 cells treated with different selenium compounds alone or with different selenium compounds combined with X-rays treatment. As shown in Figure 7E, it is obvious that the content of caspase-3 in SPC-A1 cells under the action of X-rays alone is 1.8 times that of the control group. The increase in caspase-3 when SeD is combined with X-rays is significantly higher than when the drug alone reacts, which was 3.6 times that of the control group. In conclusion, different selenium compounds can play a role in promoting tumor cell apoptosis by activating the intracellular apoptosis executive molecule caspase-3, and the activation ability SeD > SeC = Na_2_SeO_3_>LET-SeNPs.

## 4 Conclusion

**SCHEME 1:**
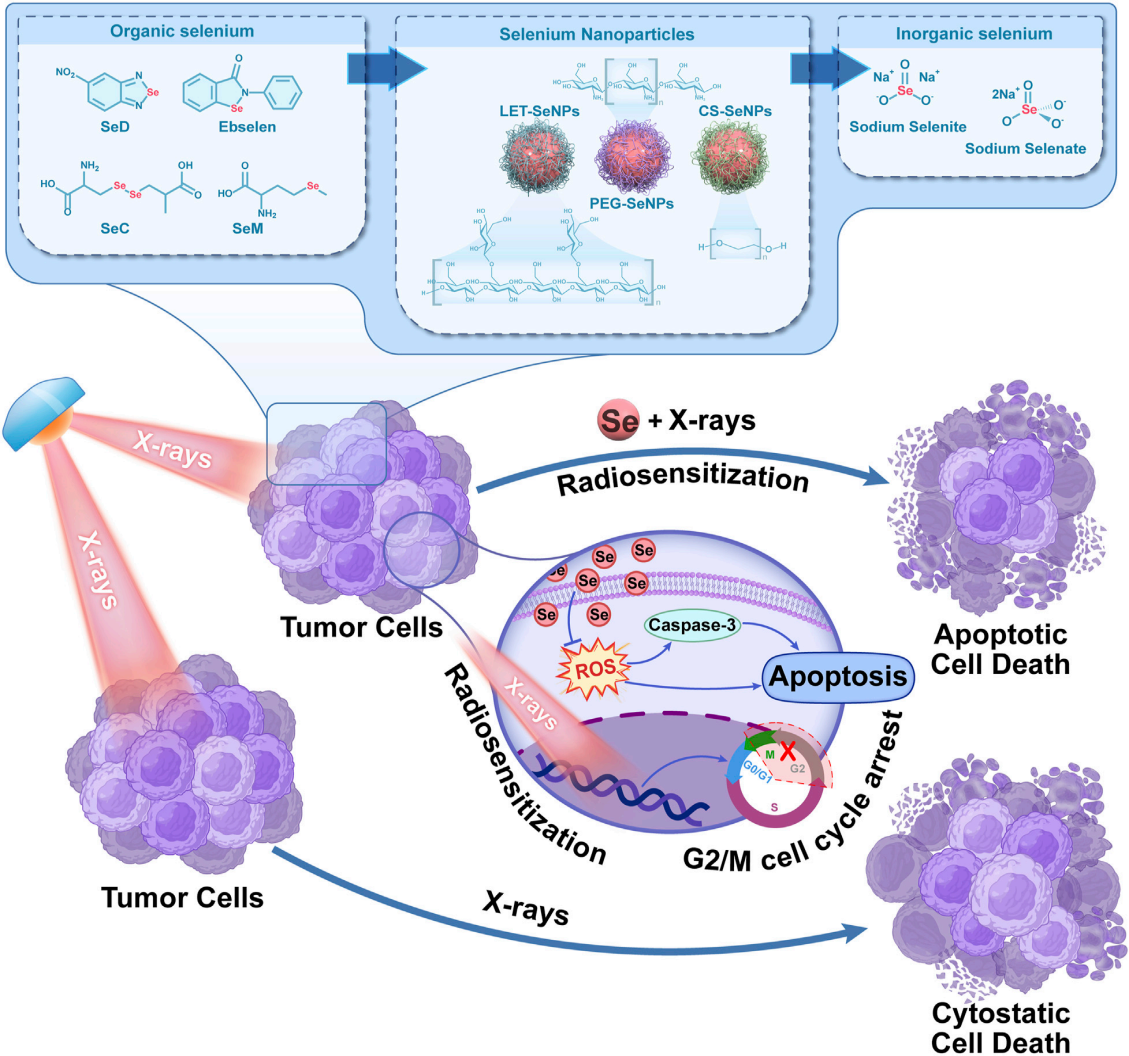
Schematic diagram of X-rays combined with selenium to inhibit the proliferation of lung cancer cells.

In conclusion, we compared the mechanism of action of selenium in different valences (inorganic selenium, organic selenium, selenium-containing amino acids and nano-selenium) on NSCLC SPC-A1 cells by X-rays alone or in combination Scheme 1. The possible ways of sensitizing radiotherapy with different selenium compounds were preliminarily explored. The results showed that selenium compounds mainly inhibited the growth and reproduction of tumor cells by blocking SPC-A1 cell cycle in G2/M phase, inducing apoptosis, causing REDOX imbalance, inhibiting injury and repair of tumor cells. Among them, -2-valent SeD showed good antitumor activity in vitro cell experiments. It mainly inhibited tumor cell proliferation and migration by enhancing X-rays at low doses, and induced SPC-A1 cell apoptosis through ROS pathway and caspase cascade reaction. Sodium selenate with +6 valence has essentially no cytotoxic effect. Therefore, this study found that the radiosensitization effect of selenium on NSCLC was closely related to the morphology of selenium. It provides a new choice for radiosensitizers for clinical NSCLC, and provides a scientific theoretical basis for further application of selenium compounds in the clinical treatment of NSCLC.

## Data Availability

The original contributions presented in the study are included in the article/Supplementary Material, further inquiries can be directed to the corresponding authors.
