# Supplementary material for: Selenium speciation-dependent cancer radiosensitization by induction of G2/M cell cycle arrest and apoptosis
Source: Front Bioeng Biotechnol. 2023 Mar 22;11:1168827. doi: 10.3389/fbioe.2023.1168827 (PMC10073679; doi:10.3389/fbioe.2023.1168827)
Supplement: Supplementary file 1 [file DataSheet1.docx]

Supporting information

for

**Selenium speciation-dependent cancer radiosensitization by induction of G2/M cell cycle arrest and apoptosis**

Shiqing Nie^#^, Xin He^#^, Zhiting Sun, Yan Zhang, Ting Liu, Tianfeng Chen*, Jianfu Zhao*

Department of Oncology of the First Affiliated Hospital, Jinan University, Guangzhou, Guangdong, China, 510632

*** Correspondence:**Email addresses: tchentf@jnu.edu.cn (T. Chen); zhaojianfu@jnu.edu.cn (J. Zhao).

# Equal contribution.

1. **Experimental section**

These selenium compounds have been synthesized and published before. All the selenium compounds and SeNPs were supplied by Dr. Tianfeng Chen’s lab in Jinan University.

**Preparation of Different Modified SeNPs**

We started by mixing 1 mL of (10 mg/mL) CS, PEG, LET storage solution and 1 mL of (10 mM) sodium selenite (Na_2_SeO_3_) storage solution in a glass bottle. And then added Vc (40 mM, 1 mL) and ultra-pure water (2 mL). The mixture was stirred at room temperature overnight and dialyzed in Milli-Q ultra-pure water for 24 h-48 h. Finally, the concentration of Se was measured by inductively coupled plasma atomic emission spectroscopy (ICP-AES).

1. **Supplementary Figures and Tables**


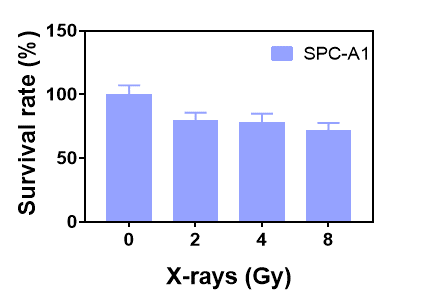


**Figure S1. SPC-A1 cells under X-rays (0–8 Gy) irradiation then incubated for another 72 h**


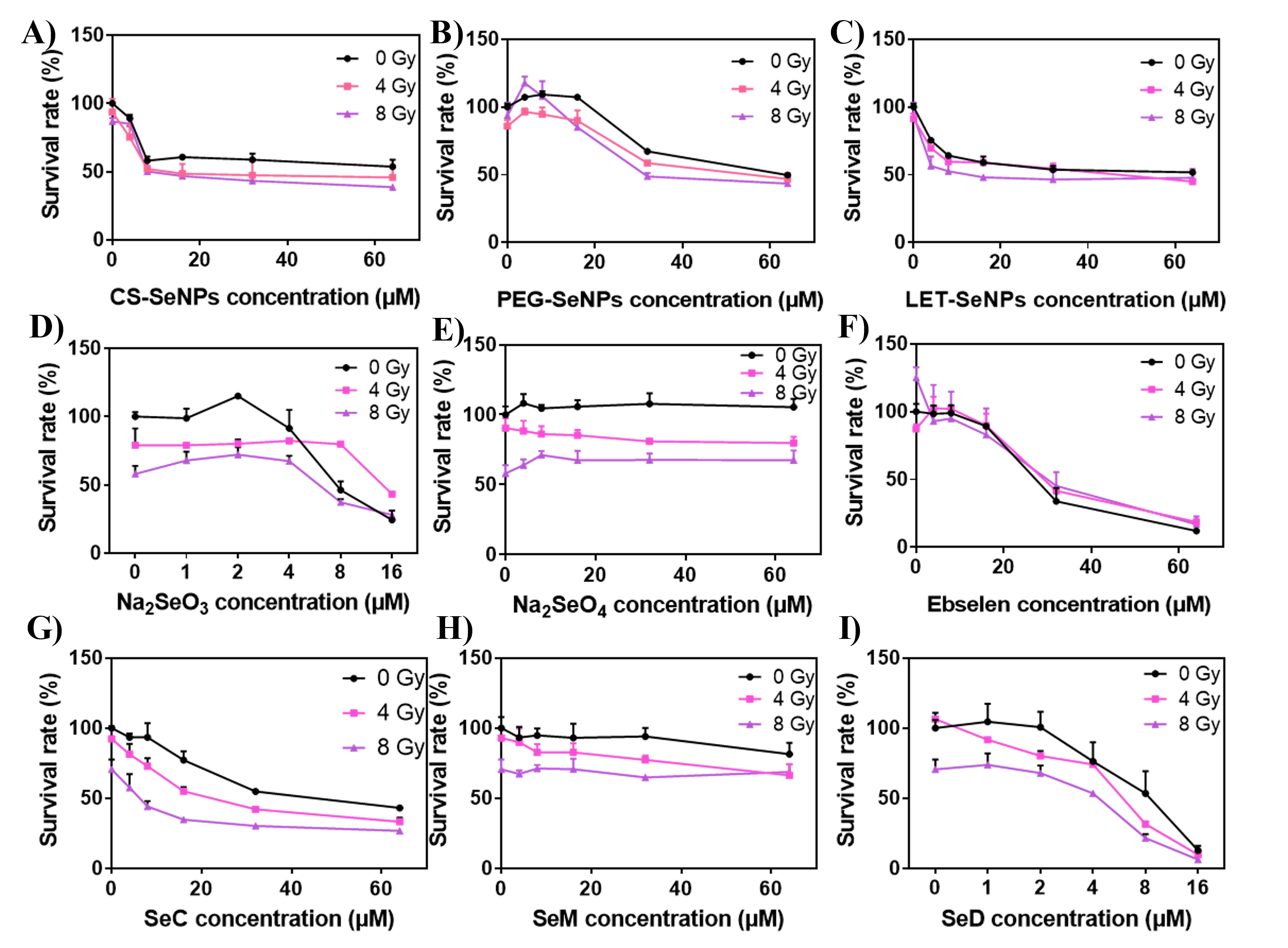


**Figure S2. Cell survival curves of different seleni um compounds combination with X-rays.** (A-I) Cell viability of SPC-A1 cells after treatment with different selenium compounds combination with X-rays for 72 h.


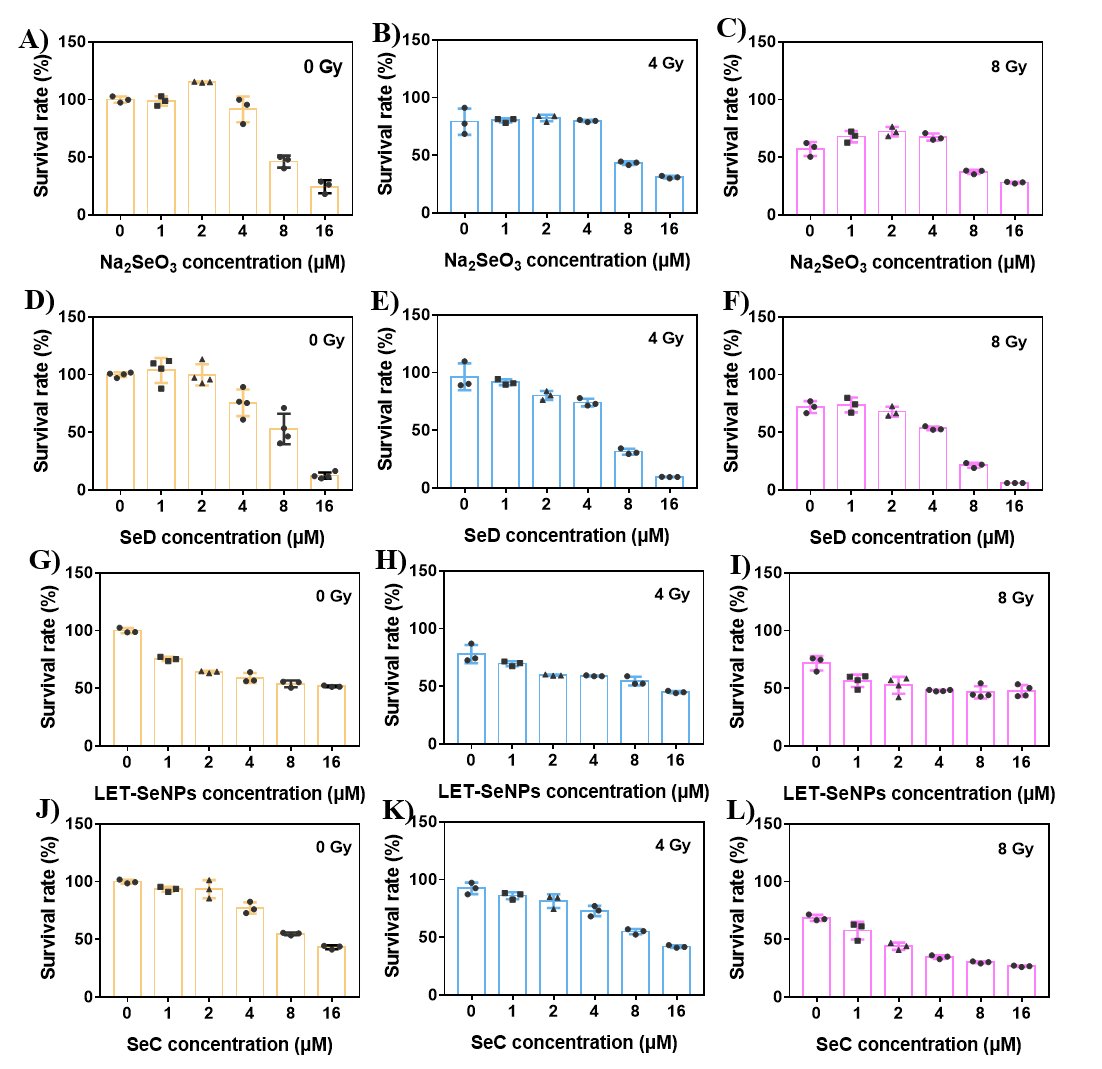


**Figure S3.** **Histogram of cell survival of different selenium compounds under X-ray action.** Cell viability after 72 h treatment of SPC-A1 cells with different concentrations of Na_2_SeO_3_ (A-C), SeD (D-F), LET-SeNPs (G-I), SeC (J-L) combined with different doses of X-ray.


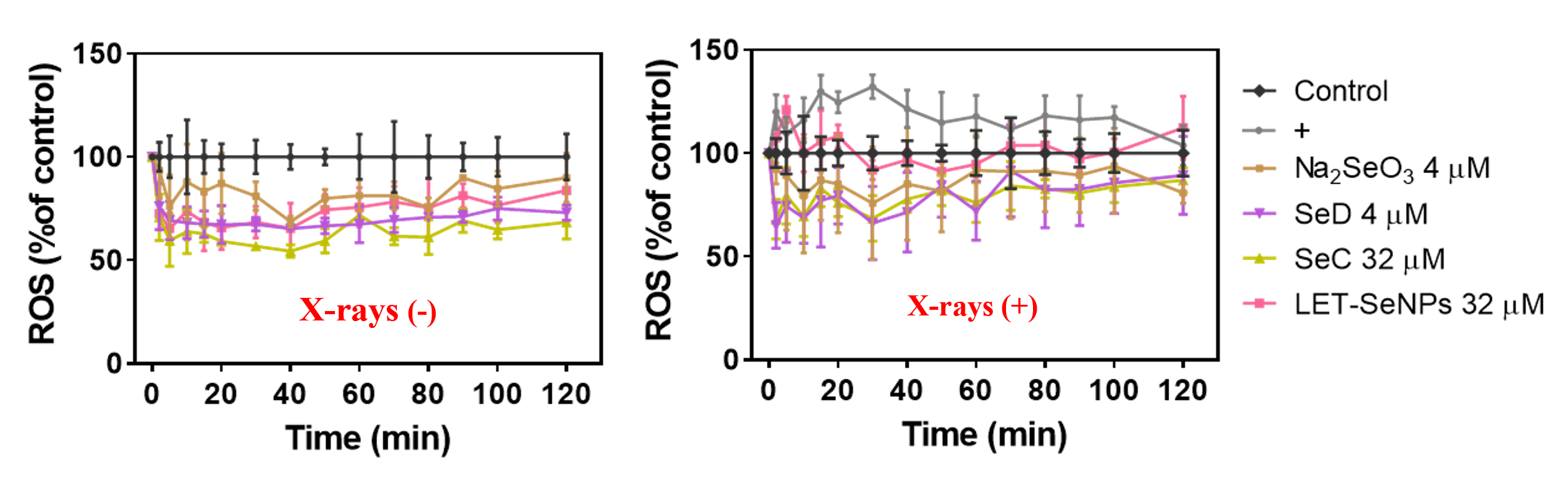


**Figure S4.** **Different selenium compounds combination with X-rays changes intracellular ROS levels.** (A) ROS changes after interaction of different selenium compounds. (B) ROS changes after interaction of different selenium compounds and X-rays.

**Table S1. Growth inhibition of different seleni um compounds radiation treatment on SPC-A1 (calculated by the Se concentration, 72 h).**

| Complex | IC_50_ (μM)^a^ | | |
| --- | --- | --- | --- |
|  | 0Gy | 4Gy | 8Gy |
| CS-SeNPs | >64 | 19.02 | 24.13 |
| PEG-SeNPs | 58.49 | 52.6 | 43.02 |
| LET-SeNPs | 58.86 | 42.23 | 17.65 |
| Na_2_SeO_3_ | 8.497 | 9.051 | 7.438 |
| Na_2_SeO_4_ | >64 | >64 | >64 |
| Ebselen | 27.37 | 30.33 | 30.33 |
| SeC | 22.49 | 21.91 | 12.77 |
| SeM | >64 | >64 | >64 |
| SeD | 7.846 | 5.27 | 3.762 |

1. Concentration for 50% cell growth inhibition.
